# The Yin and the Yang of Treatment for Chronic Hepatitis B—When to Start, When to Stop Nucleos(t)ide Analogue Therapy

**DOI:** 10.3390/v12090934

**Published:** 2020-08-25

**Authors:** Samuel Hall, Jessica Howell, Kumar Visvanathan, Alexander Thompson

**Affiliations:** 1 Gastroenterology Department, St Vincent’s Hospital Melbourne, 41 Victoria Pde, Fitzroy, VIC 3065, Australia; jessica.howell@svha.org.au (J.H.); alexander.thompson@svha.org.au (A.T.); 2 Infectious Diseases Department, St Vincent’s Hospital Melbourne, 41 Victoria Pde, Fitzroy, VIC 3065, Australia; kv@unimelb.edu.au

**Keywords:** hepatitis B virus, nucleos(t)ide analogue, cessation

## Abstract

Over 257 million individuals worldwide are chronically infected with the Hepatitis B Virus (HBV). Nucleos(t)ide analogues (NAs) are the first-line treatment option for most patients. Entecavir (ETV) and tenofovir disoproxil fumarate (TDF) are both potent, safe antiviral agents, have a high barrier to resistance, and are now off patent. They effectively suppress HBV replication to reduce the risk of cirrhosis, liver failure, and hepatocellular carcinoma (HCC). Treatment is continued long-term in most patients, as NA therapy rarely induces HBsAg loss or functional cure. Two diverging paradigms in the treatment of chronic hepatitis B have recently emerged. First, the public health focussed “treat-all” strategy, advocating for early and lifelong antiviral therapy to minimise the risk of HCC as well as the risk of HBV transmission. In LMICs, this strategy may be cost saving compared to monitoring off treatment. Second, the concept of “stopping” NA therapy in patients with HBeAg-negative disease after long-term viral suppression, a personalised treatment strategy aiming for long-term immune control and even HBsAg loss off treatment. In this manuscript, we will briefly review the current standard of care approach to the management of hepatitis B, before discussing emerging evidence to support both the “treat-all” strategy, as well as the “stop” strategy, and how they may both have a role in the management of patients with chronic hepatitis B.

## 1. Introduction

More than 257 million people, or 3.2% of the world’s population, are estimated to be living with chronic hepatitis B infection (CHB) [1,2]. Chronic hepatitis B (CHB) is an important cause of liver-related morbidity and mortality, increasing the risk of cirrhosis, liver failure, and liver cancer (hepatocellular carcinoma, HCC) [3]. Indeed, CHB is the leading cause of HCC globally. The risk of these complications can be prevented by antiviral therapy.

The current first-line therapies for CHB include the nucleot(s)ide analogues (NAs) tenofovir disoproxil fumarate (TDF), tenofovir alafenamide (TAF), and entecavir (ETV), as well as the immunomodulator pegylated interferon-α (pegIFN) [4,5]. Most patients are treated with NA therapy. The first-line NAs are highly effective, well tolerated, and have a high genetic barrier to resistance. The goal of NA treatment is to reduce hepatic inflammation and thereby reduce both fibrogenesis and oncogenesis through suppression of HBV replication. HBV cure is very rare during NA therapy with HBsAg loss rates of <1% per year [6], therefore treatment with NA is long-term.

In contrast, pegIFN therapy is finite, with the standard treatment duration being 48 weeks. HBV cure is reported following pegIFN treatment but occurs in <5% of patients. The goal of treatment is long-term viral suppression off treatment; this occurs in <25% of patients overall and is lower among patients with HBeAg-negative CHB. PegIFN is delivered by subcutaneous injection and adverse events are common, including flu-like symptoms, cytopaenias, neuropsychiatric problems, and thyroid dysfunction. PegIFN therapy is only used in a selected minority of patients due to the limited long-term efficacy and concerns about tolerability. PegIFN will not be discussed further in this review.

In international management guidelines, treatment for CHB is currently recommended for people with high HBV DNA levels in blood, in combination with evidence of hepatic necro-inflammation and/or moderate-advanced hepatic fibrosis (Table 1) [4,5,7]. Treatment is therefore targeted at those individuals with the highest risk of clinical complications, particularly cirrhosis and HCC. Nucleot(s)ide analogue treatment has been subsidised or reimbursed in many regions of the world for more than a decade, based on cost-effectiveness modelling performed at the time of the registration studies. However, TDF and ETV are now off patent, so costs of therapy have markedly reduced. Nucleot(s)ide analogues effectively prevent liver fibrosis progression and can lead to fibrosis regression long term [8].

One of the most important challenges for the field is to prevent HCC. Long-term NA therapy reduces risk but does not completely prevent HCC development [9,10,11,12,13]. Recent data suggest that oncogenesis begins early in the natural history of CHB, is driven by active viral replication and genomic integration of HBV sequence into the host genome, and is cumulative over time [10]. Data also suggest that the HCC risk reduction associated with NA therapy and HBV DNA suppression increases over time [9]. Tenofovir DF and ETV are both off patent, and generic formulations are widely available at low cost. Indeed, the annual cost of TDF is now lower than the cost of an annual HBV DNA assay in most LMICs. As noted, TDF and ETV are highly effective, well tolerated, and have a high genetic barrier to resistance. In this context, there is an emerging argument for starting NA therapy early in the immunotolerant phase of disease, to suppress HBV replication and minimise the long-term risk of HCC. This strategy is being proposed as “treatment for all” and will be explored in detail in this review.

Conversely, there is also interest in whether it is possible to withdraw long-term NA therapy in some patients with HBeAg-negative CHB. This strategy hypothesises that long-term NA therapy is associated with reconstitution of the HBV-specific immune response, which can lead to durable viral suppression off treatment. There are also data suggesting that stopping NA therapy may promote HBsAg loss [14,15,16]. Evidence to support patient selection for this “stop strategy” will be presented. Patients with HBeAg-positive CHB have a higher risk of severe hepatitis flares after stopping NA therapy in the absence of HBeAg seroconversion, and this population will not be discussed.

## 2. “Treatment for All”—A Public Health Approach to CHB

**Figure 1:**
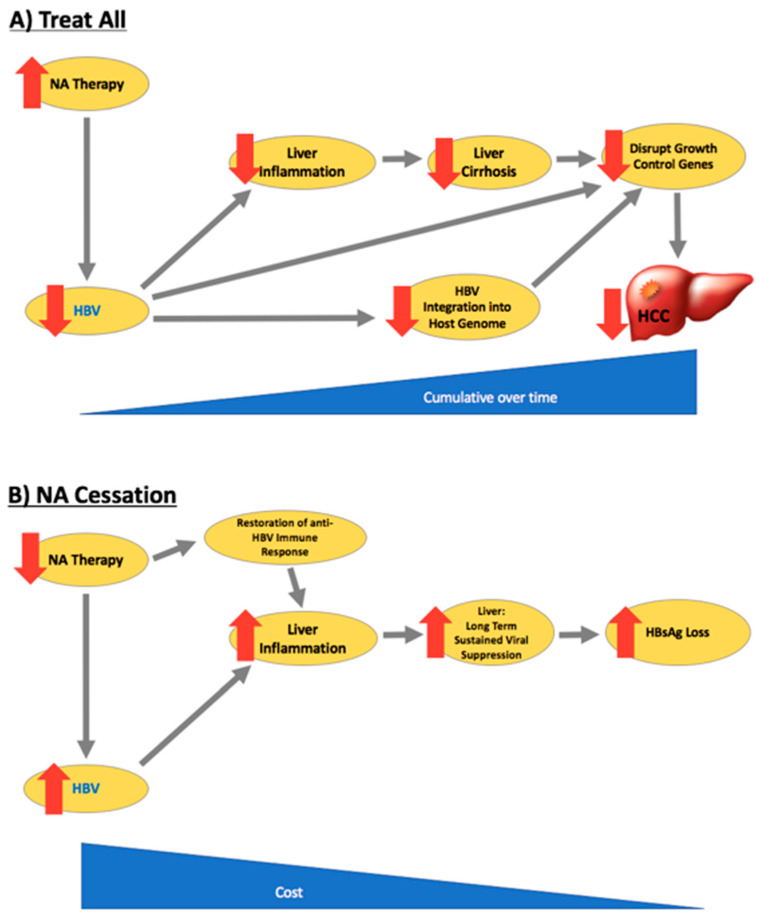
Benefits of “treat all” and nucleos(t)ide analogue (NA) cessation: HBsAg loss rates and hepatocellular carcinoma (HCC)/cirrhosis risk. (**A**) “Treat all” reduces HBV viraemia, hepatic inflammation, and thus cirrhosis and HCC risk; (**B**) “NA cessation” causes an initial HBV viral relapse with subsequent increase in rates of HBsAg loss.

Chronic hepatitis B is associated with liver-related morbidity and mortality, including cirrhosis, liver failure, and HCC [9]. Viral suppression prevents liver fibrosis progression, cirrhosis, and liver failure and reduces the risk of HCC (Figure 1) [4,17,18,19,20,21,22]. The REVEAL study of Taiwanese CHB patients was the first study to demonstrate that HBV-related HCC risk is strongly associated with HBV DNA levels in blood, and that transition from high HBV DNA levels to low HBV DNA levels over time was associated with a reduction in HCC risk [19]. Antiviral therapy has been shown in clinical trials to reduce liver necro-inflammation and fibrosis in patients with HBeAg-positive and HBeAg-negative chronic hepatitis (immune control, immune reactivation phases, respectively) and in patients with advanced fibrosis or cirrhosis, to reduce the risk of liver failure and HCC [8,18,22,23,24,25,26,27,28,29,30,31]. Current guidelines therefore recommend NA therapy for people with a high HBV DNA level and at least moderate liver fibrosis and/or inflammation [4,5,7] (Table 1). However, although NA therapy reduces HCC risk, HCC is not completely prevented. Long-term follow-up studies demonstrate a low but persistent risk of HCC in patients treated with NA (annual incidence in non-cirrhotic patients of 0.01–1.4% and in those with cirrhosis, 0.9–5.4%) [10]. Prevention of HCC is now the major clinical challenge for the field and the major cause of liver-related mortality in patients receiving antiviral therapy. The recent observation that the rate of incident HCC starts to fall after 5 years of NA therapy and that the benefit increases over time, suggests that longer duration of therapy is required to achieve maximum risk reduction. It also raises the important question of whether starting treatment earlier than currently recommended by guidelines might improve clinical outcomes. A key issue is whether NA therapy should be considered in the immunotolerant (IT) phase of CHB. The recent EASL guidelines have recently expanded treatment criteria to include people aged over 30 years with a viral load greater than 2000 IU/mL, even in the absence of significant fibrosis or a raised alanine aminotransferase (ALT) level, in recognition of the higher risk of HCC with increasing age after 30 years [4,5].

Emerging data supports treatment of patients in the IT phase of CHB. A recent Korean retrospective cohort study showed that the risk of HCC was higher among 413 HBeAg-positive IT patients who did not meet current guideline criteria, compared with 1497 immune clearance patients (HBeAg positive with high ALT) who were treated with NA [32]. Patients in the IT phase who remained untreated had a 2–3 times higher risk of HCC, liver transplantation, or death compared with patients in the immune clearance phase who received antiviral therapy. Although there are limitations to this dataset—retrospective analysis, the median age of the IT patients was 38 years, some had ALT levels >ULN, and 26% had HBV DNA levels <10^7^ IU/mL—the data suggest significant risk of HCC that might be reduced by NA therapy.

The association between viral suppression and HCC risk reduction likely occurs through two mechanisms: the prevention of cirrhosis, as well as the reduction of HBV genomic integration events. For many years, the IT phase of CHB was assumed to be benign, with high levels of viral replication, but no anti-HBV-specific immune response and no intrahepatic inflammation driving fibrogenesis [33]. However, the concept of immune tolerance itself has recently been challenged. HBV-specific T cell responses, hepatic necro-inflammatory activity [34,35], and circulating HBV variants associated with progression to HBeAg-negative disease are detectable in young IT patients [36]. The data suggest a risk of insidious intrahepatic inflammation and fibrogenesis, which might be prevented by NA therapy. Integration of HBV DNA into the host genome of infected hepatocytes is now thought to be a key event driving oncogenesis [10,37]. Genomic integration of HBV sequence can be detected frequently in young patients with IT disease and has been associated with clonal hepatocyte expansion—in fact the rate of HBV integration has been reported to be similar to that in older patients [38]. The frequency of integration events is proportional to HBV viral load, and viral suppression by NA will reduce the risk of integration events [39,40]. Liver carcinogenesis may therefore start during the IT phase of CHB, with pro-oncogenic events accumulating throughout the natural history of disease. The current treatment paradigms allow decades of oncogenic opportunity. It is a rational hypothesis that early introduction of NA therapy will reduce risk of HCC by preventing liver fibrosis progression as well as reducing the probability of viral integration in the host genome.

Broadening the current treatment criteria to include young people in the IT phase of CHB is also likely to reduce rates of horizontal and vertical transmission.

Treatment of patients in the IT phase would increase the treatment pool, with inherent cost. However, two of the first-line NAs for the treatment of CHB—ETV and TDF—are now generic, dramatically reducing their list price. The cost of generic TDF tenofovir is as low as $3 per month in some low–middle income countries—significantly less than the cost of an annual HBV DNA level using PCR technology [2]. The safety profile of both agents is very good. Long-term adverse events with ETV are very rare and toxicity monitoring is not routinely recommended. TDF has been associated rarely with renal and bone adverse events and guidelines recommend simple monitoring of patients with 6-monthly serum creatinine, eGFR, and phosphate levels (a new formulation of tenofovir, the pro-drug tenofovir alafenamide (TAF), was recently licensed that has a lower risk of renal/bony AEs compared to TDF, but remains under patent protection [23]. Therefore, the field has affordable agents suitable for long-term use that are very effective and safe.

A strategy involving “treatment for all” adults would also simplify assessment and treatment algorithms. The management of CHB is complicated: determining treatment eligibility requires multiple tests including HBV serology, HBV DNA level, serum biochemistry, and evaluation of liver fibrosis (using fibrosis biomarkers or liver histology). The natural history of CHB is variable and progressive, necessitating long-term monitoring. CHB screening and treatment strategies could be simplified to one of “test and treat”. In some regions with endemic hepatitis B, this strategy may even be cost saving—as noted, the annual costs of generic NA treatment are now lower than the costs of disease monitoring in many LMICs [2]. Even in developed economies, the current complexity requires specialist involvement, despite the fact that models of care must shift to the primary care setting to meet targets for diagnosis and treatment [1]. Significant liver fibrosis is very rare among young people in the IT phase, and NA therapy would obviate the need for ongoing liver fibrosis monitoring and may also reduce the risk of HCC to levels below which screening is cost-effective.

Adoption of this public health approach to the management of CHB could happen in two ways. Ideally, data would be collected to provide the supporting evidence base. This would require a large-scale, long-term prospective placebo-controlled study to evaluate the clinical benefit of early NA therapy for patients in the IT phase of CHB. The target population would be HBeAg-positive with high HBV DNA levels (>10^7^ IU/mL) and normal serum ALT levels. For pragmatic reasons, participants would be aged 18–30 years. The control population would be monitored according to the current standard of care guidelines and commenced on antiviral therapy at the time of progression to active hepatitis, defined by an ALT flare. The primary outcome of interest would be HCC incidence; other surrogate endpoints that would be collected, such as ALT flare, are unlikely to convince regulatory bodies to approve expansion of current treatment criteria. Cost-effectiveness would be a key secondary endpoint. It would also be important to capture qualitative data regarding patient perception of lifelong therapy starting at a young age.

The conduct of such a study will present practical challenges, including the need for a very large sample size and long follow-up period. Patient retention will be a challenge. Industry is not likely to sponsor this study given the patent positions on available NAs, making government/philanthropic funding necessary. We must also acknowledge the HBV cure strategies in active development; if these succeed, then the concept of lifelong long-term suppression will become obsolete—however, demonstration of a reduction in liver-related morbidity/mortality in an IT cohort would expand the population eligible for cure strategies. Despite these challenges, a longitudinal study evaluating the benefit of early treatment would directly inform clinical practice and guideline development. This was the approach taken for HIV, where the INSIGHT START study demonstrated the benefit of treatment initiation for all HIV-positive people regardless of CD4+ count, now a standard of care practice [41].

It may be possible to collect data without performing a traditional prospective study. In Uzbekistan, the government is working to eliminate viral hepatitis, and they are currently performing a trial to make hepatitis treatment affordable to all. All participants will receive free screening and diagnosis, and four out of five patients diagnosed with CHB will pay for treatment. This in turn will subsidise payment for the one out of five who cannot afford treatment [42]. In this model, monitoring is costlier than therapy, therefore all HBsAg-positive individuals will be treated indefinitely. The evaluation of outcomes and costs in this treat-all population could be compared to regions with treatment given according to current standard of care.

Finally, given the practical challenges to collecting prospective data, is it possible for guidelines to expand treatment indications ahead of the evidence? The recent update to the European guidelines recommending that treatment be considered for people with persistent IT CHB over the age of 30 years is a recent example of this. We would strongly advocate for prospective studies to justify changes to policy but note that clinical care can at times move ahead of the data where there is compelling rationale.

In summary, it is logical that the risk of HBV-related oncogenesis starts from the time of infection, is cumulative, and driven by HBV genomic integration events associated with high-level viral replication, as well as progressive fibrotic liver damage. The hypothesis is that early viral suppression will minimise the risk of HCC by reducing integration events and preventing cirrhosis. Treatment for all would simplify clinical management algorithms, promoting public health interventions for control of CHB, and in an era of generic NA and high-cost PCR technology, is likely to be cost effective. There is a need to define the benefit of a “treat-all” approach for IT patients. The challenge for the field is how best to do this.

## 3. “The Stop Strategy”—A Personalised Approach to NA Therapy for HBeAg-Negative CHB

In contrast to the “treat-all” strategy, the field is also exploring whether some patients taking long-term NA therapy may be able to successfully withdraw antiviral treatment. The current standard of care for HBeAg-negative chronic hepatitis B is long-term NA therapy. Endpoints for treatment are not well defined. There is consensus among international guidelines that HBsAg loss is an important end-point (Table 2) [4,5,7], but HBsAg loss is very rare during NA therapy. The Asia-Pacific guidelines were the first to consider that NA treatment might also be stopped in HBsAg-positive patients, recommending that “in patients without liver cirrhosis … treatment can be withdrawn after treatment for at least 2 years with undetectable HBV DNA documented on three separate occasions, 6 months apart” [7]. This statement was driven at the time more by economic considerations than a strong evidence base—in some regions, NA treatment is reimbursed for a finite duration. However, over the past 5–10 years the field has developed great interest in whether NA therapy can be safely withdrawn in a subset of patients.

In 2012, Hadziyannis et al. published a landmark study evaluating clinical outcomes after treatment cessation in a cohort of patients with HBeAg-negative CHB who were long-term responders to adefovir monotherapy [14]. Thirty-three HBeAg-negative CHB patients had treatment withdrawn after 4/5 years of adefovir monotherapy. All patients were from Athens and were infected with genotype D HBV. No patient was cirrhotic at the time of starting adefovir. All patients experienced early virological relapse within one month of treatment discontinuation; HBV DNA levels peaked in the first month post-treatment in 66% and in the second month in 21%. Virological relapse was associated with biochemical relapse (ALT > 1.2 × upper limit of normal) in 25 (76%) of 33 patients; the ALT rise occurred concomitantly with the virological relapse in 44%, or within 1–2 months later. Antiviral therapy was resumed in 15/25 patients after biochemical relapse. Eighteen patients remained off treatment through five years of follow-up, and all achieved a sustained response, defined by HBV DNA <2000 IU/mL and normal ALT. In what was a striking observation, 13 of the 18 (72%) patients who maintained a sustained response off treatment achieved HBsAg loss at the end of 5 years of follow-up. Virological relapse with persistent viremia led to re-commencement of NA therapy in 15 (45%) of 33 patients, but no liver decompensation events were observed. Only one of 15 patients restarted on NA achieved HBsAg loss.

Since this initial study, there have been multiple reports of clinical outcomes after stopping long-term NA therapy in HBeAg-negative patients (Table 3). The studies have been heterogeneous, with retrospective and prospective design, and there has been considerable variation between protocols for ethnicity of cohort, inclusion/exclusion of people with cirrhosis, duration of follow-up, as well as criteria for re-starting NA therapy. Some studies have included patients who were HBeAg-positive at the time NA therapy was started. Despite the heterogeneity, a number of comments can be made. Virological relapse is very common early after stopping NA, if not universal (Table 3). The rate and degree of serum HBV DNA rise varies considerably. Biochemical flare may follow virological relapse, particularly in patients in with higher HBV DNA levels (>10^4–5^ IU/mL in most studies), where severe ALT flares may occur. For this reason, withdrawal of NA therapy is not recommended for patients with cirrhosis. Virological flare may however be associated with achievement of sustained virological and biochemical response in approximately 43–67% of patients at 1–2 years [43], and as noted, some patients may achieve HBsAg loss. Most studies suggest that HBsAg loss occurs late and have described an incremental rise in HBsAg loss rates over time, with the highest reported to date of 54.9% at 6 years [16]. Rates of re-treatment vary considerably according to protocol, with indications for re-starting NA including viral relapse, biochemical relapse, severe hepatitis flare, as well as at clinician discretion. The optimal indication for re-treatment is not yet clear—early re-treatment for clinical relapse may in fact reduce the chance of HBsAg loss [15].

The overall safety of the strategy is acceptable—severe biochemical flares have been reported in some studies, but liver decompensation events are rare, and deaths have only been reported amongst HBeAg-negative patients who were cirrhotic [29]. Therefore, this is not a suitable strategy for patients with cirrhosis. It is also important to emphasise that this is a strategy that requires close monitoring after treatment withdrawal—severe hepatitis flares may occur and require timely restarting of NA therapy. As a minimum, we recommend monitoring liver function tests at week 6, week 12, week 18, and week 24, and 3 monthly thereafter for the first 2 years. Ideally monitoring should involve measurement of HBV DNA levels, but the cost of monitoring HBV DNA levels is high and, in non-cirrhotic patient, is not necessary at every visit. Patients should be educated about the symptoms of acute hepatitis. There are few reports of incident HCC in these cohorts. In one retrospective study from Taiwan, there was no significant difference in cumulative rates of HCC after stopping NAs compared to the preceding on-treatment period (annual, 3-year, and 6-year cumulative HCC incidence rates were 0.15%, 1%, and 1% compared to annual incidence and 3-year cumulative incidence rates of 0.083% and 0.3% on treatment) [15]. Despite this, we would recommend restarting NA therapy in patients with persistent HBV DNA levels >2000 IU/mL after stopping NA therapy, regardless of ALT level, to minimise the risk of HCC over time. We do not recommend repeated trials of withdrawal of HBV therapy.

It would be clinically useful to be able to identify a subset of non-cirrhotic, HBeAg-negative, HBsAg-positive patients who are more suitable for a trial of stopping NA treatment. Longer duration of NA therapy, ETV vs. TDF therapy, lower levels of HBsAg, HBV core-related antigen (HBcrAg), and HBV RNA, and higher anti-HBc levels are all end-of-treatment factors that have been associated with lower risk of relapse after stopping NAs [15,16,44,45,46,47,48,49,50,51,52,53,54]. The pre-treatment HBV DNA level has been associated with risk of relapse. After stopping, the rate of rise, as well as the peak, of HBV DNA level, has been associated with the risk of hepatitis flare. There may be differences in the rate of HBsAg loss between Western and Eastern populations, with higher rates of HBsAg loss reported in the West; whether this relates to differences in ethnicity, HBV genotype, or duration of infection is not clear. However, to date, there are no validated clinical/translational markers that have sufficient clinical utility to influence clinical decision making. Large, prospective clinical trials are needed to develop and validate clinical algorithms for a personalised approach to NA treatment withdrawal.

In summary, there have been multiple studies in the last decade that have evaluated clinical outcomes after stopping NA treatment. There appears to be a subset of patients who maintain long-term virological remission and even achieve HBsAg loss (Figure 1). We believe that withdrawal of NA treatment can be considered on an individual basis and is reasonable to consider in motivated, non-cirrhotic patients with hepatitis B mono-infection. Close monitoring is required after stopping NA treatment. This is not a strategy for people with cirrhosis, for people who are not motivated to adhere to close monitoring, or for people with HIV or HDV coinfection [82,83]. The validation of biomarkers that are associated with clinical outcomes and have sufficient utility for clinical decision making will promote the development of clinical algorithms for a personalised approach to treatment in the future.

## 4. Future Directions

Currently, the goal of treatment for CHB is long-term viral suppression to reduce the risk of liver-related morbidity and mortality, including HCC. HBsAg loss is rare and hard to predict. The development of curative treatments for HBV infection would be a major advance for the field. There are multiple antiviral and immunomodulatory treatment candidates in pre-clinical and clinical development [84]. Whether these approaches succeed, and if successful what the time to market will be, is hard to predict but likely at least 5–10 years away. Therefore, there is still the need to explore strategies to improve clinical care with currently available treatments. Demonstrating the clinical benefit and cost effectiveness of starting treatment for all patients in the immunotolerant phase of disease would have important implications for practice and public health policy. It would also support universal implementation of future HBV cure strategies. The development of a long-acting HBV antiviral suitable for use as a depot preparation—as recently done for HIV [85,86]—might promote long-term compliance with a treat-all strategy. On the flip-side, further study of biomarkers to identify patients most likely to benefit from NA treatment cessation, as well as the underlying biological mechanisms predicting for sustained viral remission and HBsAg loss, will promote individualised therapy, and also potentially identify novel therapeutic strategies. In this context, there is a strong rationale for evaluating both the yin and the yang of “start” and “stop” strategies for chronic hepatitis B.

## Figures and Tables

**Table 1:** International guidelines for treating chronic hepatitis B (CHB) e-antigen negative disease.

Guidelines	Decision to Treat
APASL 2015 [7]	(i) ALT > 2 × ULN and HBV DNA > 2000 IU/mL or (ii) Moderate to severe hepatic inflammation or significant fibrosis ^a^
EASL 2017 [5]	(i) ALT > ULN and HBV DNA > 2000 IU/mL or (ii) Moderate liver necroinflammation or fibrosisor or (iii) ≥30 y, HBeAg positive, normal ALT, HBV DNA > 20,000 IU/mL, regardless of severity of liver histology (immune tolerant)
AASLD 2018 [4]	(i) ALT > 2 × ULN ^b^ and HBV DNA > 2000 IU/mL or (ii) Moderate to severe necroinflammation or fibrosis ^a^ or (iii) ≥40 y, HBeAg positive, normal ALT, HBV DNA > 1,000,000 IU/mL, liver biopsy specimen showing significant necroinflammation or fibrosis (immune tolerant)

^a^ Moderate to severe hepatic inflammation on liver biopsy mean METAVIR activity score of A2-3; significant fibrosis means ≥F2 by METAVIR fibrosis score; ^b^ AASLD definition of ULN is an ALT of 35 IU/L for males and 25 IU/L for females; EASL and APASL definition is >40 IU/L for both genders in patients with chronic hepatitis B infection. HBV: hepatitis B virus; ALT: alanine aminotransferase.

**Table 2:** Recommended endpoints for NA treatment in HBeAg-negative CHB.

EASL 2017 [5]	AASLD 2018 [4]	APASL 2015 [7]
HBsAg loss	HBsAg loss	HBsAg loss
“Discontinuation of NAs in selected non-cirrhotic HBeAg-negative patients who have achieved long-term (3 years) virological suppression under NA(s) may be considered if close post-NA monitoring can be guaranteed”	-	“In patients without liver cirrhosis…treatment can be withdrawn after treatment for at least 2 years with undetectable HBV DNA documented on three separate occasions, 6 months apart”

**Table 3:** Off-therapy outcomes post cessation of NAs in patients with HBeAg-negative CHB.

Reference	*n*	Median F/U (m)	% Clinical Relapse (EOF)	% HBsAg Loss (EOF)	% NA Restart (EOF)	Notes Prospective/Retrospective % Asian/Caucasian HBeAg + Included Cirrhosis Included Safety If Significant Events
Berg et al. [43]	21	33	58 ^1,a^	19	38; Re-started for CR	RCT 4.8% Asian/95.2% Caucasian
Cao et al. [55]	22	21	53 ^a^	0	NR	HBeAg ± cohort
Chen et al. [48]	169	20	52 ^1,a^	7.7	39; Re-start criteria NR	Retrospective Study HBeAg ± and cirrhosis 2% HCC (EOF) 3% Hepatic Decompensation (EOF) 0.4% OLT (EOF)
Ge et al. [56]	204	24	NR	0.5	NR	Retrospective Study
Ha et al. [57]	145	16	NR	8.3	61; Re-start criteria NR	
Hadziyannis [14]	33	66	76 ^1,b^	39	45; Re-started for CR	100% Caucasian Adefovir only included
He et al. [58]	66	17	NR	0	NR	Retrospective Study
Hsu et al. [59]	133	13	39 ^1,a^	0.75	NR	3% hepatic decompensation in non-cirrhotic cohort
Hung et al. [60]	73	67	NR	27	52; Re-started for VR	Only Cirrhosis included
Jeng et al. [45]	95	12	45 ^1,a^	0	36; Re-start criteria NR	Retrospective-Prospective Study Cirrhosis included
Jung et al. [61]	68	30	28 ^1,a^	0	40; Re-start criteria NR	Cirrhosis included
Kang et al. [62]	60	67	10 ^2,a^	18.3	25; Re-start criteria NR	HBeAg ± and cirrhosis 5% HCC (EOF)
Kim et al. [63]	45	26	53 at 12 m* (EOF NR) ^1,a^	0	NR	Cirrhosis included
Lee et al. [64]	37	22	35 ^2,b^	5	NR	HBeAg ± cohort
Liu et al. [65]	61	15	NR	10.2	61; Re-start criteria NR	
Pan et al. [66]	30	115	77 ^b^	9.3	NR	Retrospective-Prospective Study and HBeAg ± cohort
Patwardhan et al. [67]	33	36	48 ^1,b^	0	48; Re-start criteria NR	Retrospective Study Ethnicity NR
Peng et al. [68]	21	12	33 ^1,a^	1.5	27; Re-started for CR	HBeAg ± cohort
Seto et al. [46]	184	12	NR	0	91; Re-started for VR	Cirrhosis included
Sohn et al. [69] ^‡^	54	22	NR	0	67; Re-started for VR	Retrospective Study HBeAg ± and cirrhosis NA restarted if HBV Detectable
Wang et al. [70]	46	25	59 ^1,a^	0	58; Re-start criteria NR	HBeAg ± cohort
Yao et al. [16]	119	60	27.6 ^1,a^	54.9	24; Re-started for CR	Cirrhosis included
Chen et al. [54]	104	13	49 ^1,a^	6	33; Re-started for CR	Retrospective Study HBeAg ± cohort 2% Hepatic Decompensation (EOF)
Chen et al. [71]	263	12	53 ^1,a^	13	42; Re-start criteria NR	HBeAg ± cohort 0.5% HCC (EOF)
Jeng et al. [15]	691	36	61 ^1,a^	6	41; (Re-start Criteria: ‘discretion of the treating physician’)	Retrospective-Prospective Study; Cirrhosis included 691 HBeAg − patients (0 HBeAg + patients) Hepatic Decompensation Annual Incidence Rates: 0% in non-cirrhosis and 2.95% in cirrhosis; Mortality rates: 1% died in Cirrhosis and 0% in non-cirrhosis; HCC Annual Incidence Rates: 0.083% in non-cirrhotic and 1.52% for cirrhosis
Kuo et al. [50]	353	25	54 ^1,a^	8	44; Re-started for CR	Retrospective-Prospective Study; HBeAg ± cohort 1.16% HCC (EOF) 2.7% Hepatic Decompensation (EOF)
Liem et al. [72]	41	17	73 ^1,b^	2.2	38; Re-start criteria NR	RCT 98% Asian/2% Caucasian HBeAg ± cohort
Liu et al. [73]	85	60	NR	14	NR	HBeAg ± cohort 1.6% HCC (EOF)
Ma et al. [74]	375	12	55 ^1,a^	1	55; Re-started for CR	HBeAg ± cohort 19% Hepatic Decompensation (EOF) 0.2% Mortality rate (EOF)
Papatheodoridis et al. [75]	130	15	55 ^3,b^	0	33; Re-started for CR	41.5% Asian/58.5% Caucasian Definition of Virological Relapse (HBV DNA > 60 IU/mL) 0.8% HCC (EOF)
Papatheodoridis et al. [76]	57	18	43 ^3,b^	21	28; Re-started for CR	Ethnicity NR Definition of Virological Relapse (HBV DNA > 60 IU/mL) 1.8% HCC (EOF
Su et al. [77]	72	34	45 ^1,a^	0	40; Re-started for CR	HBeAg ± cohort
Chi et al. [78]	59	19	NR	38	38; Re-started for VR	79.7% Asian/20.3% Caucasian HBeAg ± cohort and cirrhosis included Hepatic Decompensation: 0% in non-cirrhosis and 2% in cirrhosis
Hsu et al. [79]	124	17	25.8 ^1,a^	6	NR	HBeAg ± cohort
Lee et al. [80]	44	21	32 ^1,a^	0	42; Re-start criteria NR	HBeAg ± cohort and cirrhosis included
Lee et al. [81]	93	12	41.9 ^1,a^	0	NR	Retrospective Study

^‡^ patients were restarted on NA therapy if HBV DNA became detectable; virological relapse definitions: HBV DNA ^1^ >2000 IU/mL, ^2^ >1000 cpm, ^3^ >60 IU/mL; clinical relapse definitions: ^a^ ALT > 2 × ULN, ^b^ ALT > ULN; NR = not reported; EOF = end of follow-up; clinical relapse (CR); virological relapse (VR); OLT = orthotopic liver transplantation; m* = months; note that they are prospective Asian studies unless otherwise indicated.
